# Retrospective Evaluation of Patients With Scorpion Stings Admitted to the Pediatric Emergency Clinic

**DOI:** 10.7759/cureus.29606

**Published:** 2022-09-26

**Authors:** Feyat Tunç, Süleyman Yıldız, Mehmet Celal Devecioglu, İlyas Yolbas, Fesih Aktar

**Affiliations:** 1 Pediatrics and Child Health, Batman Training and Research Hospital, Batman, TUR; 2 Pediatrics and Child Health, Mardin Derik State Hospital, Mardin, TUR; 3 Pediatrics and Child Health, Dicle University Faculty of Medicine, Diyarbakır, TUR; 4 Pediatrics and Child Health, Dicle University Faculty of Medicine, Diyarbakir, TUR

**Keywords:** pediatric emergency, mortality, prazosin, antivenom, scorpion sting, children

## Abstract

Introduction

Scorpion sting in children is still a serious health problem today. Children are at greater risk of developing severe cardiac, respiratory, and neurological complications because of their low body weight. In this study, we retrospectively evaluated the demographical changes, complaints, clinical findings, and laboratory results of scorpion sting cases admitted to the pediatric emergency department.

Materials and Methods

The records of 72 patients who were followed up with the diagnosis of scorpion sting in the Dicle University Pediatric Emergency Department between 2013 and 2017 were retrospectively analyzed.

Results

The patients included in the study were between one and 15 years (7.64±4.04 years) and 43.1% were male, and 56.9% were female. While 65.3% of the cases lived in rural areas, 34.7% lived in the city center. The most common stung areas in the cases were the lower extremity (51.4%) and the upper extremity (34.7%). The most common complaints in the patients were 70.8% pain, 58.3% edema, 41.7% cold extremities, 23.6% sweating, 22.2% vomiting, and 12.5% excessive salivation. Of the cases, 71.4% had mild, 25.7% had moderate, and 2.9% had severe stages. Of the patients, 91.6% were given antivenom, 75.7% were given antihistamines, 74.3% were given steroids, 65.7% were given antibiotics, 64.3% were given analgesics, 44.3% were given tetanus vaccine, 2.8% were given erythrocyte suspension and 1.4% were given platelet suspension. In addition, 11.4% of the cases were given prazosin treatment. While 32.9% of the cases required intensive care, two patients died. A statistically significant difference was found between the glucose, urea, creatine, total protein, sodium, potassium, alanine aminotransferase, white blood cell count, red blood cell count, hemoglobin, hematocrit, neutrophil count values of the patients at admission and discharge.

Conclusion

Scorpion sting cases are still a significant health problem. The severe clinical course is more common in children. The management of patients with severe clinical forms is based on early recognition of the sting, antivenom serum administration, and cardiorespiratory and systemic support.

## Introduction

Poisoning after scorpion sting and snake bites is one of the critical pediatric emergencies that can result in death in childhood [1,2]. Scorpion stings are still a common health problem in Turkey, especially in the southeastern Anatolia region like Mardin, Batman, and Diyarbakir, where hot seasons occur [3]. Although poisoning does not always occur due to species differences in scorpion stings, life-threatening findings may develop more quickly, especially in children, due to the high amount of toxins exposed per kilogram. Patients' complaints start within the first five hours after a scorpion sting and usually end within one to two days [2]. The first complaint is generally related to the serotonin in the venom [4]. Systemic effects occur due to the release of acetylcholine and catecholamines [5]. While findings such as hyperthermia, tachycardia, tachypnea, hypertension, hyperglycemia, and pulmonary edema may be seen due to the effect of the sympathetic system, bronchoconstriction, bradycardia, hypotension, increased secretion in the whole body, and myosis may be seen due to parasympathetic effect [6]. The generally accepted treatment is symptomatic treatment and antivenom administration [1]. Patients with systemic symptoms should be monitored and treated in the intensive care unit [7,8].

## Materials and methods

The records of 72 patients who were followed up with the diagnosis of scorpion sting in the Pediatric Emergency Department of Dicle University between 2013 and 2017 were retrospectively examined. Ethics committee approval was obtained from Dicle University Ethics Committee with a decision no. 25.01.2018/54. Patients' general characteristics (age, gender), epidemiological data (location, bite site), hospital admission complaints, physical examination, laboratory findings, and treatments they received (such as tetanus vaccine, scorpion antivenom, and antihistaminic therapy), complications, and prognosis were evaluated. The patients were evaluated regarding vital signs, body temperature, respiratory rate, blood pressure, pulse rate, respiratory and neurological conditions, hematological and biochemical parameters, systemic and local findings during the monitoring period.

Statistical Analysis

The data obtained from this study were evaluated in the SPSS 15.0 for Windows program (SPSS Inc., Chicago). The quality evaluation, program entry, and analysis of the data were made by the researcher himself. Student's t-test and one-way analysis of variance (ANOVA) test were used in cases where parametric conditions were met. The Mann-Whitney U test and Kruskall-Wallis test were used in cases where parametric conditions were not met in the analyses where the clinical and laboratory levels were compared with various variables. The statistical significance level was accepted as p<0.05.

## Results

This study consisted of 72 scorpion stings. According to the study's demographic results and general characteristics, 56.9% of the patients were female and 43.1% were male. The patients' ages ranged from one to 15 years (7.64±4.04 years). 65.3% of the cases were in a rural area, and 34.7% were in the city center. 51.4% of the patients were stung by scorpions from the lower extremity and 34.7% were stung from the upper extremity (Table 1).

**Table 1:** Distribution of patients according to demographic and general characteristics

	n	%
Sex		
Male	31	43.1
Female	41	56.9
Age		
1-3 years	15	20.8
4-6 years	14	19.4
7-9 years	18	25.0
10-12 years	16	22.2
13-15 years	9	12.5
Region/Area lived in		
City center	25	34.7
Rural	47	65.3
Sting area		
Upper extremity	25	34.7
Lower extremity	37	51.4
Head-Neck	3	4.2
Other states	7	9.7

While 23 (32%) of the patients needed intensive care, two (2.7%) of these patients died during treatment in the intensive care unit. The most common clinical signs and symptoms were pain in 70.8%, vomiting in 22.2%, edema in 58.3%, cold extremities in 41.7%, and sweating in 23.6% (Table 2).

**Table 2:** Clinical signs and symptoms of the patients at the time of admission

	n	%
Pain	51	70.8
Edema	42	58.3
Cold Extremity	30	41.7
Sweating	17	23.6
Vomiting	16	22.2
Excessive Salivation	9	12.5
Convulsion	5	6.9
Priapism	1	1.4

The most common treatments applied to the cases were antivenom (91.6%), antihistamine (76.4%), steroid (75%), antibiotic treatment (66.7%), analgesic (65.3%), and tetanus vaccine (44.4%) (Table 3).

**Table 3:** Treatments administered to the cases

	n	%
Antivenom administered		
Intramuscular	14	19.4
Intravenous	52	72.2
Antivenom application method		
Single dose administered	40	55.6
Multiple-dose administered	10	13.9
Tetanus vaccine administered	32	44.4
Steroid administered	54	75
Given antihistamines	55	76.4
Starting antibiotic treatment	48	66.7
Given erythrocyte suspension	2	2.8
Administered platelet suspension	1	1.4
Fresh frozen plasma delivered	2	2.8
Using prazosin	8	11.1
Using dopamine	7	9.7

When the laboratory values of the patients during admission and discharge were compared, there was a statistically significant difference between blood glucose, urea, creatine, total protein, sodium, potassium, alanine aminotransferase (ALT), number of white blood cells (WBC), number of red blood cells (RBC), hemoglobin (HGB), hematocrit (HCT), and neutrophil count values (Table 4).

**Table 4:** Laboratory values of the patients at the time of admission and before discharge ALT: Alanine Aminotransferase, AST: Aspartate Aminotransferase, LDH: Lactate dehydrogenase, CK: Creatine Kinase, Ca: Calcium, Na: Sodium, K: potassium, CRP: C-reactive protein, CK-MB: Creatine phosphokinase-2, WBC: White blood cell count, RBC: Red blood cell count, HGB: Hemoglobin count, HCT: Hematocrit

	Value at the time of admission			Pre-discharge value			p
	n	Mean±	SD	n	Mean	SD	
Glucose (mg/dl)	70	117.66±	48.53	70	98.43±	19.32	0.002
Urea (g/dL)	70	24.59±	8.60	70	20.97±	7.54	0.000
Creatine (mg/dl)	70	0.57±	0.12	70	0.51±	0.10	0.000
Total protein (U/L)	70	7.15±	0.64	70	6.78±	0.71	0.000
Albumin (gr/dl)	70	4.05±	0.38	70	4.27±	3.86	0.632
Total bilirubin (mg/dl)	70	0.40±	0.48	70	0.39±	0.25	0.749
ALT (U/L)	70	18.27±	8.35	70	23.02±	20.68	0.048
AST (U/L)	70	32.83±	15.55	70	34.56±	24.71	0.557
LDH (U/L)	70	305.90±	69.98	70	315.47±	265.35	0.760
CK (U/L)	70	296.93±	400.30	70	256.60±	540.70	0.570
Ca (mmol/L)	70	10.87±	11.36	70	22.05±	107.56	0.391
Na (mmol/L)	70	138.37±	4.34	70	136.90±	2,17	0.004
K (mmol/L)	70	4.07±	0.75	70	4.30±	0.44	0.025
CRP (mg/dl)	65	0.15±	0.24	65	0.15±	0.25	0.873
CK-MB (ng/mL)	70	8.34±	12.77	70	6.67±	11.80	0.343
Troponin (ng/mL)	70	0.21±	1.11	70	0.04±	0.14	0.178
WBC (10^3^/uL)	70	11.84±	4.72	70	9.70±	3.68	0.001
RBC (10^6^/µLl)	70	4.94±	0.38	70	4.78±	0.44	0.000
HGB (g/Dl)	70	12.94±	1.22	70	12.51±	1.27	0.000
HCT (%)	70	37.80±	5.12	70	35.96±	6.45	0.002
Platelets (10^3^/uL)	70	321.22±	86.69	70	308.71±	89.04	0.127
Lymphocyte (10^3^/uL)	70	2.81±	1.87	70	3.10±	1.58	0.237
Monocyte ((10^3^/uL)	70	0.55±	0.33	70	0.59±	0.26	0.275
Neutrophil ((10^3^/uL)	65	8.01±	3.83	65	5.40±	3.01	0.000
Eosinophil (10^3^/uL)	70	0.20±	0.25	70	0.26±	0.28	0.076

## Discussion

Scorpion sting is a critical health problem in children, especially those under five years of age [9]. Since children cannot protect themselves against environmental hazards and their awareness levels are lower than adults, their morbidity and mortality levels may be significantly higher. Therefore, unlike adults, the child age group can be stung by scorpions from more than one body part [3].

The variable age distribution is observed in scorpion stings in other studies [2,3]. In a study conducted by Osnaya-Romero et al., the most common admission to the hospital was seen in patients aged one-three years, and the average admission age was 5.2 years [10]. Adiguzel et al. observed that children between the ages of nine and 15 (54.1%) were more exposed to stings than other age groups [11]. In addition to the more studies showing that the frequency of hospital admission is more common in females, there are also studies showing that it is higher in males. [12-15]. In this study, scorpion stings were more common in women. It was observed that the patients were mostly between seven to nine years of age.

Clinical symptoms in scorpion stings can vary from mild to severe, and the most common findings include restlessness, cold extremities, tachycardia, and hypotension [3,16]. Scorpions can sting when they are accidentally contacted or feel in danger. Considering the body parts stung by scorpions, the extremities are the most common in many studies [17]. They can also sting the patient neck or head area during sleep time [2]. In this study, the most common clinical symptoms were pain in 70.8%, edema in 58.3%, cold extremities in 41.7%, sweating in 23.6%, and vomiting in 22.2%. Furthermore, the arms and legs were the most affected areas of the body (86.1%). These results are similar to previous studies in the literature [16-18]

Depending on the effect of toxins, abnormal hematological values (leukocytosis, thrombocytopenia), renal (increased urea and creatinine), liver [increased ALT and aspartate aminotransferase (AST)], cardiac (tachycardia, bradycardia, ST-T changes), and pulmonary involvement can be seen [3,6]. In our study, when the control blood values of the patients were compared during admission and discharge, glucose, urea, creatine, total protein, ALT, Na (sodium), K (potassium), WBC, RBC, hemoglobin, hematocrit, and neutrophil values were found to be significantly higher. Hyperglycemia is one of the poor prognostic factors identified in scorpion stings. Additionally, hyperglycemia and hypokalemia can cause electrocardiographic changes. In the study by Çağlar et al., patients with high glucose values at admission showed a worse prognosis [8,19]. In this study, the blood glucose values at admission were higher than the control blood glucose values before discharge.

Various methods can be used in the treatment of scorpion stings. In our study, it is understood that treatment methods were applied similarly to the literature. Prazosin shows its effect by antagonizing the ability to stimulate the specific ion channel of scorpion toxins. The death rate due to scorpion stings has decreased below 1% after using prazosin, which has been used since 1984 [2,20,21]. Our hospital's case-fatality rate fell to 3% in children due to scorpion stings after prazosin was used as the first-line treatment method [2].

Single-center data and a retrospective study design are the main limitations of this study. The other limitations in this study include the lack of some laboratory parameters and there are deficiencies in some anamnesis.

## Conclusions

Scorpion envenomation is an important public health issue, and life-threatening complications can be seen more frequently in pediatric patients. Some blood parameters, like hyperglycemia on admission, may be warning signs for severe patients. Early treatment and intensive care support are of great importance in such cases. Initiating antivenom and/or prazosin treatment with early symptomatic treatment in patients with systemic symptoms is vital to reduce mortality and morbidity. Further studies are needed to define the effect of different management modalities in childhood scorpion envenomation.

## References

[1] Oto A, Haspolat YK (2021). Venomous snakebites in children in Southeast Turkey. Dicle Med J.

[2] Bosnak M, Levent Yilmaz H, Ece A (2009). Severe scorpion envenomation in children: management in pediatric intensive care unit. Hum Exp Toxicol.

[3] Bosnak M, Ece A, Yolbas I, Bosnak V, Kaplan M, Gurkan F (2009). Scorpion sting envenomation in children in southeast Turkey. Wilderness Environ Med.

[4] Uluğ M, Yaman Y, Yapici F, Can-Uluğ N (2012). Scorpion envenomation in children: an analysis of 99 cases. Turk J Pediatr.

[5] Karnad DR (1998). Haemodynamic patterns in patients with scorpion envenomation. Heart.

[6] Doğanay Z, Karatas AD, Baydın A (2006). Is administration of antivenin necessary for all cases of scorpion envenomation? A case report. Türkiye Acil Tıp Dergisi.

[7] Biswal N, Mathai B, Bhatia BD (1993). Scorpion sting envenomation: complications and management. Indian Pediatr.

[8] Çağlar A, Köse H, Babayiğit A, Öner T, Duman M (2015). Predictive factors for determining the clinical severity of pediatric scorpion envenomation cases in Southeastern Turkey. Wilderness Environ Med.

[9] Gümüştekin M (2003). Environmental toxins: animal bites and stings (Article in Turkish). Türkiye Klinikleri Toksikoloji Özel.

[10] Osnaya-Romero N, de Jesus Medina-Hernández T, Flores-Hernández SS (2001). Clinical symptoms observed in children envenomated by scorpion stings, at the children's hospital from the State of Morelos, Mexico. Toxicon.

[11] Adiguzel S, Ozkan O, Inceoglu B (2007). Epidemiological and clinical characteristics of scorpionism in children in Sanliurfa, Turkey. Toxicon.

[12] Tekin R, Sula B, Cakırca G (2015). Comparison of snakebite cases in children and adults. Eur Rev Med Pharmacol Sci.

[13] Forrester MB, Stanley SK (2004). Epidemiology of scorpion envenomations in Texas. Vet Hum Toxicol.

[14] Pardal PP, Castro LC, Jennings E, Pardal JS, Monteiro MR (2003). [Epidemiological and clinical aspects of scorpion envenomation in the region of Santarém, Pará, Brazil]. Rev Soc Bras Med Trop.

[15] Al-Asmari AK, Al-Saif AA (2004). Scorpion sting syndrome in a general hospital in Saudi Arabia. Saudi Med J.

[16] Biswal N, Bashir RA, Murmu UC, Mathai B, Balachander J, Srinivasan S (2006). Outcome of scorpion sting envenomation after a protocol guided therapy. Indian J Pediatr.

[17] Bawaskar HS (1977). Scorpion sting and cardiovascular complications. Indian Heart J.

[18] Abourazzak S, Achour S, El Argam L (2009). Epidemiological and clinical characteristics of scorpion stings in children in Fez, Morocco. J Venom Anim Toxins incl Trop Dis.

[19] Lira-da-Silva RM, Amorim AM, Brazil TK (2000). Poisonous sting by Tityus stigmurus (Scorpiones; Buthidae) in the state of Bahia, Brazil (Article in Portuguese). Rev Soc Bras Med Trop.

[20] Al-Sadoon MK, Jarrar BM (2003). Epidemiological study of scorpion stings in Saudi Arabia between 1993 and 1997. J Venom Anim Toxins incl Trop Dis.

[21] de Roodt AR, Garcia SI, Salomon OD (2003). Epidemiological and clinical aspects of scorpionism by Tityus trivittatus in Argentina. Toxicon.

